# Upregulation of Tissue Factor by Activated Stat3 Contributes to Malignant Pleural Effusion Generation via Enhancing Tumor Metastasis and Vascular Permeability in Lung Adenocarcinoma

**DOI:** 10.1371/journal.pone.0075287

**Published:** 2013-09-27

**Authors:** Hsuan-Heng Yeh, Wen-Tsan Chang, Kuang-Chu Lu, Wu-Wei Lai, Hsiao-Sheng Liu, Wu-Chou Su

**Affiliations:** 1 Institute of Basic Medical Sciences, College of Medicine, National Cheng Kung University, Tainan, Taiwan; 2 Cancer Center, National Cheng Kung University Hospital, College of Medicine, National Cheng Kung University, Tainan, Taiwan; 3 Department of Biochemistry and Molecular Biology, College of Medicine, National Cheng Kung University, Tainan, Taiwan; 4 Department of Surgery, National Cheng Kung University Hospital, College of Medicine, National Cheng Kung University, Tainan, Taiwan; 5 Department of Microbiology and Immunology, College of Medicine, National Cheng Kung University, Tainan, Taiwan; 6 Department of Internal Medicine, National Cheng Kung University Hospital, College of Medicine, National Cheng Kung University, Tainan, Taiwan; 7 Center for Infectious Disease and Signaling Research, National Cheng Kung University, Tainan, Taiwan; Cincinnati Children's Hospital Medical Center, United States of America

## Abstract

Malignant pleural effusion (MPE) is a poor prognostic sign for patients with lung cancer. Tissue factor (TF) is a coagulation factor that participates in angiogenesis and vascular permeability and is abundant in MPE. We previously demonstrated that autocrine IL-6-activated Stat3 contributes to tumor metastasis and upregulation of VEGF, resulting in the generation of MPE in lung adenocarcinoma. In this study, we found IL-6-triggered Stat3 activation also induces TF expression. By using pharmacologic inhibitors, it was shown that JAK2 kinase, but not Src kinase, contributed to autocrine IL-6-induced TF expression. Inhibition of Stat3 activation by dominant negative Stat3 (S3D) in lung adenocarcinoma suppressed TF-induced coagulation, anchorage-independent growth *in vitro*, and tumor growth *in vivo*. Consistently, knockdown of TF expression by siRNA resulted in a reduction of anchorage-independent growth of lung adenocarcinoma cells. Inhibition of TF expression also decreased the adhesion ability of cancer cells in normal lung tissues. In the nude mouse model, both lung metastasis and MPE generation were decreased when PC14PE6/AS2-siTF cells (TF expression was silenced) were intravenously injected. PC14PE6/AS2-siTF cells also produced less malignant ascites through inhibition of vascular permeability. In summary, we showed that TF expression plays a pivotal role in the pathogenesis of MPE generation via regulating of tumor metastasis and vascular permeability in lung adenocarcinoma bearing activated Stat3.

## Introduction

Lung cancer is the leading cause of cancer death in the world, including Taiwan. At the time of initial diagnosis, around 15% of lung cancer patients have pleural effusion, while 50% of the patients develop pleural effusion at later stages [1,2]. Lung cancer patients with malignant pleural effusion (MPE) have inferior prognosis, therefore, this group of patients are reclassified as early metastasis (M1a)-Stage 4 instead of stage 3B according to the current American Joint Committee on Cancer (AJCC) staging guidelines. Patients with MPE have poor quality of life and a short life expectancy; however, there is no specific treatment for MPE [3]. Therefore, understanding the molecular mechanism of MPE may shed light on developing better treatment options.

Tissue factor, a transmembrane receptor protein, is the principal physiological initiator of blood coagulation [4]. TF interacts with coagulation factor VIIa (FVIIa), triggering a cascade of proteolytic events to induce clot formation. In addition to inducing coagulation, the TF/FVIIa complex also turns on G protein-coupled protease-activated receptor (PAR) intracellular signals [5]. The signals induced by TF are known to promote tumor progression and increase angiogenesis [6-8]. Aberrant TF expression has been detected in a variety of human tumors, including glioma, breast cancer, leukemia, colon cancer, pancreatic cancer, and non-small cell lung cancer, but is not found in corresponding normal tissues [9]. TF expression levels also correlates with clinical tumor progression[10-12]. Studies have demonstrated that TF also plays an important role in tumor metastasis. Tumor cells expressing TF can result in increased metastatic potential, and inhibition of TF reduces the metastasis [13-15].

Angiogenesis and pleural vascular hyperpermeability are believed to mediate the formation of pleural effusion [16]. High levels of angiogenic factors, inflammatory cytokines, chemokines, and coagulation factors are detected in MPE [17-19]. Among them, vascular endothelial growth factor (VEGF) is a key mediator of increased pleural vascular permeability for generating MPE [16]. Furthermore, tumor necrosis factor-α (TNF-α augments additional proangiogenic and propermeability effects via upregulating VEGF expression in the pleural cavities of patients with MPE [20]. The contribution of autocrine IL-6-activated Stat3 to MPE in lung adenocarcinoma is also through VEGF upregulation [21]. Blockage of monocyte chemotactic protein-1 (MCP-1) or angiopoietin impairs MPE formation in immunocompetent mice [22,23]. In the same model, the decreased formation of MPE by inhibition of tumor NF-κB is through a mechanism other than altered VEGF or MCP-1 production [24]. Therefore, there are multiple potential pathways leading to MPE. Further studies to delineate the interaction is warranted.

High levels of TF are detected in MPE of human lung cancer patients [17]. However, it is not known whether TF plays a crucial role in the formation of MPE. The TF:FVIIa complex expressed on cancer cell surfaces activates coagulation to enhance permeability of the tumor microenvironment [25]. We have found that Stat3 activated by autocrine IL-6 mediates the generation of malignant effusion via upregulation of VEGF in lung adenocarcinoma [21]. Based on these observations and the properties of TF on increasing vascular permeability [26], it is possible that TF is one of the downstream targets of IL-6/Stat3 signaling, which contributes to the pathogenesis of lung adenocarcinoma and MPE.

In this study, we revealed that autocrine IL-6-induced Stat3 activation could upregulate TF expression. Elevated TF expression participated in MPE generation via its promotion of tumor metastasis and increase in vascular permeability in lung adenocarcinoma bearing activated Stat3.

## Materials and Methods

### Materials

The active-form Stat3 (Stat3C) plasmid was kindly provided by Dr. James Darnell, Jr. [27]. The mammalian expression plasmid for the dominant-negative mutants of Stat3, Stat3D, was kindly provided by Dr. Toshio Hirano [28]. A previously described TF siRNA sequence was used [29] and constructed into the psiVec vector, producing the plasmid psiTF. The TF reporter gene was kindly provided by Dr. Nigel Mackman [30]. The pharmacologic inhibitors AG490, PP2, and PP3 were obtained from Biomol International L.P. (Plymouth Meeting, PA). INC424 was purchased from Selleck Chemicals (Houston, TX). Stat3 (# F-2), Src (# B-12), JAK2 (#M126), and pJAK2-Y1007/1008 (#sc-16566) antibodies were obtained from Santa Cruz Biotechnology, Inc. (Santa Cruz, CA). Tissue factor (#4503) antibody was obtained from American Diagnostica (Stamford, CT). Monoclonal anti-HA (# HA-7), β-actin (# AC-40), and anti-Flag M2 (#M2) antibodies were obtained from Sigma-Aldrich (St Louis, MO). pStat3-Y705 (#9131)- and pSrc-Y416 (#2101)-specific antibodies were obtained from New England Biolabs (Beverly, MA).

### Cell lines

The PC14PE6/AS2 cell line was established from ascites generated by PC14PE6 in a SCID mouse as described previously [22]. CL 1-0 and CL 1-5 cells were kindly provided by Dr. Pan-Chyr Yang (National Taiwan University, Taipei, Taiwan) [31]. H1650 cells were obtained from American Type Culture Collection (ATCC; Rockville, MD). Individual clones stably expressing active-form Stat3 (Stat3C) or dominant-negative Stat3 (Stat3D) in PC14PE6/AS2 cells were established by transfection of plasmids and selection using geneticin (G418) (500 µg/ml). Individual clones stably expressing TF siRNA (psiTF) or vector control (psiVec) in PC14PE6/AS2 cells were established by co-transfection with the pRcCMV plasmid, which contains a neomycin resistance gene and is selected using geneticin.

### Soft agar assay

Six-well trays (Falcon, Franklin Lakes, NJ) were layered with 1 ml of 0.6% basal agar dissolved in DMEM plus 10% serum before use. Trypsinized cell suspension (1×10^4^ cells/0.1 ml) was added to 0.9 ml of 0.33% agar dissolved in DMEM plus 10% serum at 39°C. After gentle mixing, the cells were seeded into the six-well trays. After two weeks, colonies were stained with 0.05% crystal violet solution, photographed and counted.

### Animals and models

BALB/c nude mice (6-8 weeks old) were obtained from the National Laboratory Animal Center (Nangang, Taipei, Taiwan) and kept under specific pathogen-free conditions in the Animal Center of National Cheng Kung University, Taiwan. The experimental protocol adhered to the regulation of the Animal Protection Act of Taiwan and was approved by the Laboratory Animal Care and Use Committee of the University (IACUC Approval No. 100188). For the solid tumor model, cells (1×10^6^) suspended in 0.1 ml 1XPBS were subcutaneously injected into each BALB/c nude mice. The tumor volume was calculated according to the formula V = 0.52 × a^2^ × b (a, smallest superficial diameter; b, largest superficial diameter). For the experimental lung metastases and pleural effusion model, cells (1×10^6^) suspended in 0.1 ml 1XPBS were injected through the tail vein of BALB/c nude mice. The mice were sacrificed 26 days after injection and the lung lesions and pleural effusion were evaluated. For the ascites model, cells (1×10^6^) were injected into the peritoneal cavities of nude mice. The amount of ascites in the mice was evaluated on day 21.

### Cell lysis and Western blot analysis

Total cellular protein lysates were prepared as described previously [32]. Briefly, cells were washed twice with ice-cold PBS, scraped, and centrifuged. Cell pellets were lysed with whole-cell extract lysis buffer (Tris 50 mM pH 7.4, NP-40 1%, EDTA 2 mM, NaCl 100 mM, Na orthovanadate 10 mM, 0.1% sodium dodecyl sulfate (SDS), leupeptin (10 mg/ml), aprotinin (2 mg/ml), and phenylmethylsulfonyl fluoride 100 mM) (protease inhibitors from Roche Applied Science, Indianapolis, IN). Fifty micrograms of whole-cell extract were separated by SDS-polyacrylamide gels and transferred to a nitrocellulose filter (Millipore, Billerica, MA) using an electroblotter (Amersham Pharmacia Biotech Inc., Piscataway, NJ). After blocking with PBS buffer containing 5% nonfat milk, membranes were incubated with specific antibodies. Binding of each antibody was detected using an electrochemiluminescence kit (Amersham) according to the manufacturer’s instructions.

### RNA extraction and semiquantitative RT-PCR

TRIzol reagent (Invitrogen Corp., Carlsbad, CA) was utilized to extract total RNA. For RT-PCR, first-strand cDNA was synthesized from 0.2-1 µg of total RNA with an oligo-dT primer and the Moloney murine leukemia virus (MMLV) reverse transcriptase (Promega, Madison, WI). The sequences of PCR primers were as follows: tissue factor sense primer, 5'-ATC TCG CCG CCA ACT GGT AG-3'; tissue factor antisense primer, 5'-GCT GTC TGT ACT CTT CCG GT-3'; GAPDH sense primer, 5'-GAC CAC AGT CCA TGC CAT CAC-3'; and GAPDH antisense primer, 5'-GTC CAC CAC CCT GTT GCT GTA-3'. The PCR protocol was performed with the tissue factor primers at 94°C for 30 s, 55°C for 30 s, and 72°C for 1 min (35 cycles), followed by 72°C for 10 min. The PCR protocol was performed with the GAPDH primers at 94°C for 30 s, 55°C for 30 s, and 72°C for 1 min (25 cycles), followed by 72°C for 10 min. PCR products were resolved on 1.5% agarose gel.

### Luciferase reporter assays

Triplicate samples of 1 × 10^5^ cells in 35-mm plates were transfected using Lipofectin (GIBCO BRL, Life Technologies, Inc., Grand Island, NY). Three micrograms of tissue factor reporter gene (pTF-LUC-2) and 0.5 µg of β-galactosidase expression vector were co-transfected with 2 µg of pRcCMV (vector control) or 2 µg of pRcCMV-Stat3C (active-form Stat3) into PC14PE6/AS2 cells. The medium was changed to fresh DMEM 6 h after transfection. The cells were then incubated for 24 h, and luciferase and β-gal activities were determined using a luciferase assay system (Promega, Madison, WI). Luciferase activities were normalized with respect to β-gal activities.

### H&E staining

The tumor specimens were fixed in 10% formalin/PBS, dehydrated, and embedded in paraffin wax. Paraffin blocks were cut into 4-µm-thick sections. Sections were stained with H&E (hematoxylin and eosin) and examined with light microscopy.

### Immunofluorescence staining

Cells were fixed with 4% PBS-buffered paraformaldehyde for 20 min at room temperature. After cells were washed three times in PBS without permeabilization, they were incubated with TF monoclonal antibody for 1 h. Cells were then washed and incubated with Alexa Fluor 488-conjugated goat anti-mouse antibody. The nuclei were visualized by 4,6-diamidino-2-phenylindole staining (DAPI, Sigma-Aldrich, Saint Louis, MO). The immunofluorescence images were detected by confocal microscopy (Olympus, FV-1000).

### Flow cytometry assay

To determine cell surface TF expression, cells were fixed with 4% formaldehyde in PBS at room temperature for 10 min. After cells were washed twice with PBS without permeabilization, they were stained with rabbit anti-mouse TF antibody or control normal mouse IgG (1 µg/ml) for 1 h at room temperature and then incubated with Alexa Fluor 488-conjugated goat anti-mouse IgG. Following this, cells were analyzed using flow cytometry (FACSCalibur; Becton Dickinson).

### TF activity assay

Cells were harvested and lysed in 50 mM Tris-buffered saline (pH 8.0) with 1% Triton-X-100. After centrifuging, the supernatant protein was quantified and subjected to TF activity measurement using an AssaySense TF Chromogenic Activity Assay kit (Assaypro, St. Charles, MO) according to the manufacturer’s instruction. Briefly, cell lysates were incubated with coagulation factor VII (FVII) and X (FX) at 37°C for 30 min. FXa substrate was then added and the absorbance was read at 405 nm. The standard curve based on standards supplemented by the manufacturer was used to evaluate TF activity.

### In Vivo adhesion assay (Stamper-Woodruff assay)

PC14PE6/AS2-siTF and PC14PE6/AS2-siVec cells stably expressing green fluorescent protein (GFP) were applied (1 × 10^6^/100 µl) to 10 µm thick frozen lung sections on glass slides with shake at 70 rpm for 20 min. Cells were washed with PBS to remove unbound cells and then fixed in 2% glutaraldehyde for 15 min. The adhering cells were counted under a fluorescence microscope at 20X magnification in 3 random fields.

### Miles assay

The Miles assay was performed as described previously [21]. Briefly, the mice were intravenously injected with 200 µl of 0.5% Evans blue dye. After 10 min, serum-free culture supernatants of tumor cells (10^6^ cells/48 h in 50 µl of MEM) were injected intradermally on the dorsal skin of the nude mice. After 30 min, the mice were sacrificed and their skin removed and photographed.

### Statistical analysis

Statistical analysis was performed using Prism4 (GraphPad Software for Science Inc., San Diego, CA). Results were expressed as the mean ± standard error of the mean (SEM). Statistical significance was determined at values of P < 0.05. Differences between two independent groups were determined using the Mann-Whitney U test.

## Results

### Expression of TF was induced by autocrine IL-6 through a JAK2-dependent pathway in lung adenocarcinoma cells

**Figure 1:**
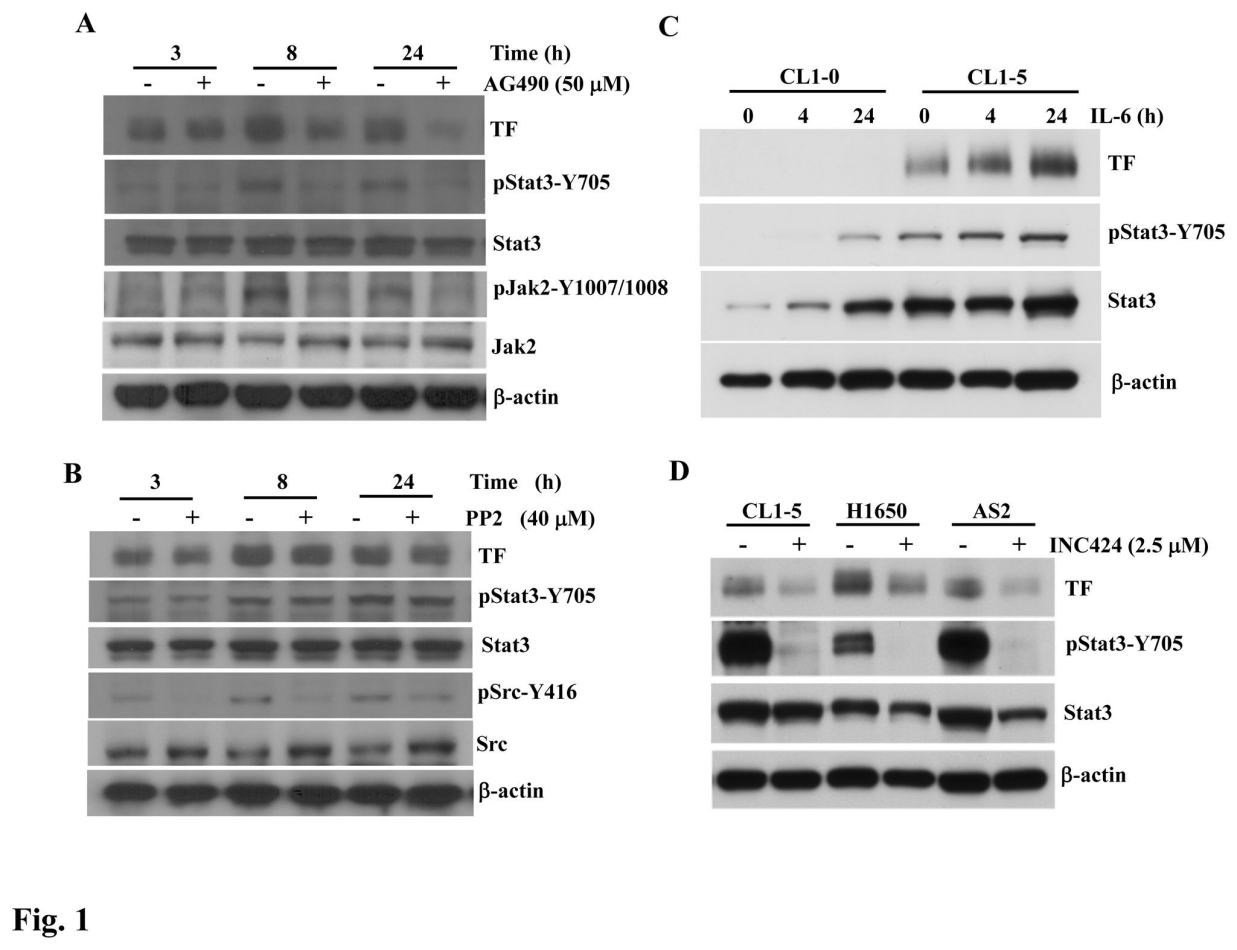
Autocrine IL-6-induced TF expression through JAK2/Stat3 signaling in lung adenocarcinoma cancer cells. (A) PC14PE6/AS2 cells, after seeding for 24 h, were incubated in the serum-free medium without (−) or with (+) AG490 (50 µM) for the indicated times. Cell lysates were analyzed by Western blot analysis using various antibodies as indicated. (B) IL-6 levels in the culture medium of PC14PE6/AS2 cells after being serum-starved for 0.5, 3, 8, and 24 h were measured using ELISA. (C) CL 1-0 and CL 1-5 cells were treated with IL-6 (10 ng/ml) for various time points. The cells were harvested and subjected to analysis of Stat3 activation and TF expression by Western blot. (D) CL 1-5, H1650 and AS2 lung adenocarcinoma cells were treated with the JAK inhibitor, INC424 (2.5 µM), for 24 h. Inhibition of Stat3 activation and TF expression were analyzed by Western blot.

Because autocrine IL-6 in PC14PE6/AS2 cells induces Stat3 activity through a JAK2-dependent signaling pathway to regulate tumor metastasis and the formation of malignant pleural effusion (MPE) [21], the purpose of this study was to clarify whether IL-6/JAK2/Stat3 signaling can induce TF expression in lung adenocarcinoma cells and whether TF expression affects the above-mentioned biological functions. TF expression, without AG490 (JAK inhibitor) treatment, was gradually elevated in PC14PE6/AS2 cells after serum deprivation of various durations (Figure 1A). The expression of TF was compatible with Stat3 phosphorylation induced by autocrine IL-6 through JAK2 activation [21]. Treatment of the PC14PE6/AS2 cells with AG490 resulted in inhibition of the phosphorylated JAK2 and Stat3 induced by autocrine IL-6 (Figure 1A). The level of TF expression induced by autocrine IL-6 was further suppressed by AG490 over time. Conversely, while treating the same cells with the Src inhibitor, PP2, the phosphorylation of Src was inhibited, whereas the levels of Stat3 phosphorylation and TF protein were unaffected (Figure 1B). PP3 serves as the control for PP2, demonstrating the specificity of the inhibitory effect on Src activation, which had no suppressive effect on Stat3 phosphorylation or TF protein expression (Figure S1). To further confirm that IL-6 induced Stat3 activation contributes to TF expression in other lung adenocarcinoma cells, CL 1-0 and CL 1-5 cells were treated with IL-6 at various time points. CL 1-0 and CL 1-5 cells are isogenetic. CL 1-5, a derivative of CL 1-0, is highly metastatic, which was established via a selection from invasive ability [31]. CL 1-5 cells with higher Stat3 phosphorylation showed more TF expression than those of CL 1-0 cells (Figure 1C). Moreover, TF expression increased gradually after IL-6 stimulation in CL 1-5 cells, compatible with Stat3 phosphorylation induced by IL-6. The Stat3 phosphorylation was also inhibited by INC424 (JAK inhibitor) in CL 1-5, H1650 and PC14PE6/AS2 (AS2) lung adenocarcinoma cells; accordingly, TF expression was decreased in those cells (Figure 1D). Together, our data revealed that autocrine IL-6 could activate Stat3 and increase the level of TF expression in various lung adenocarcinoma cell lines. Furthermore, JAK2 participated in TF expression through Stat3 activation, but in a Src-independent manner.

### TF expression was upregulated by Stat3 activation

**Figure 2:**
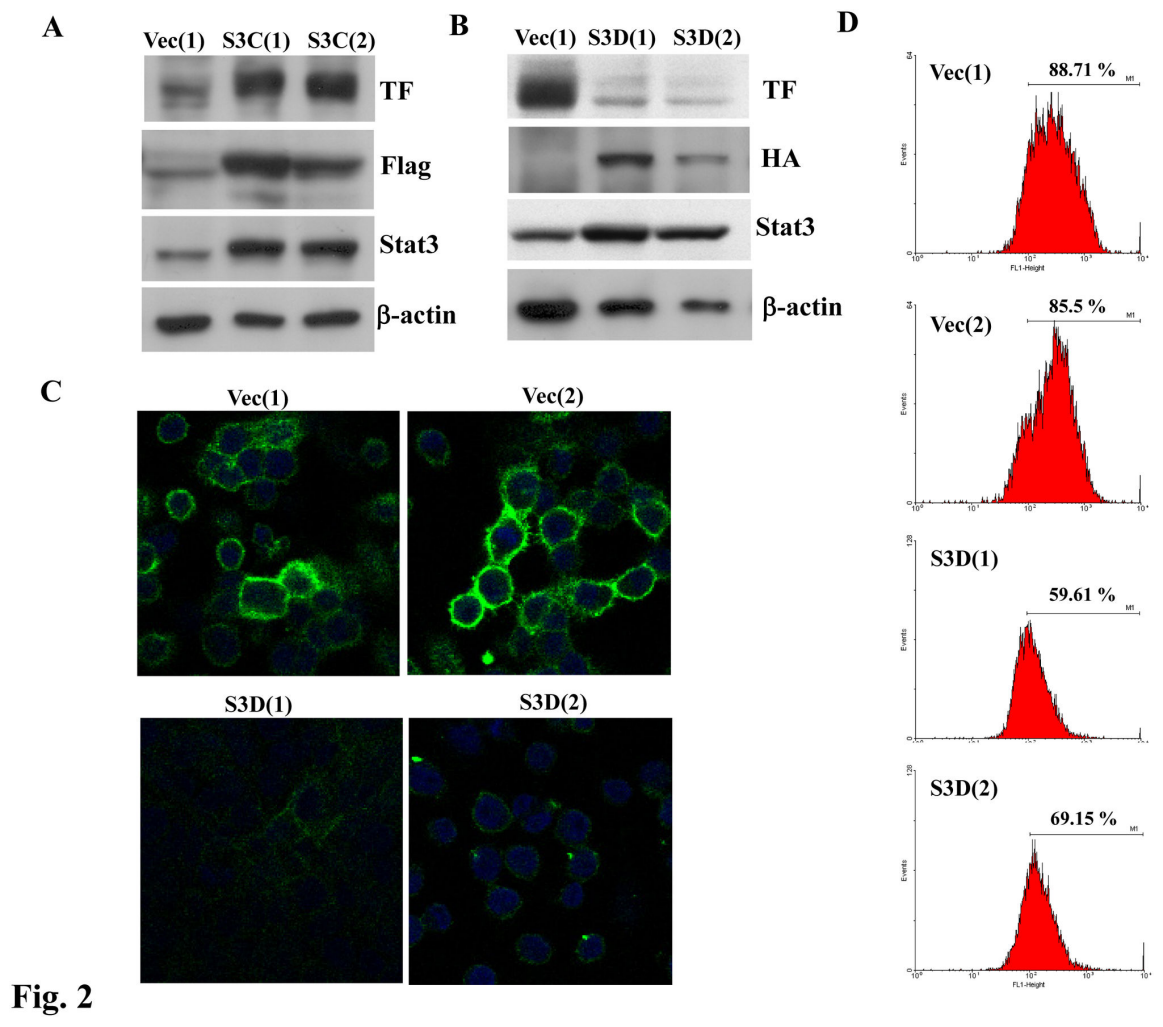
Stat3 activation increased TF expression on cell surface in PC14PE6/AS2 cells. Cell lysates of PC14PE6/AS2-Vec(1), S3C(1), and S3C(2) (A) and cell lysates of Vec(1), S3D(1), and S3D(2) (B) were harvested and subjected to Western blot analysis to detect the expression levels of Stat3 and TF protein using various antibodies as indicated. (C) Vec (1), Vec(2), S3D(1), and S3D(2) cells were seeded on chamber slides for 24 h. To visualize cell surface TF expression, the cells were fixed without permeabilization and then stained with anti-TF antibody. Images were collected by confocal microscopy. (D) Vec(1), Vec(2), S3D(1), and S3D(2) cells were seeded on a 100 mm dish for 24 h. Cells were then fixed and stained with anti-TF antibody. Signals were analyzed by flow cytometry.

To further confirm TF expression is regulated by Stat3, we established various PC14PE6/AS2 cell lines stably expressing active or dominant negative forms of Stat3 to examine the expression of TF protein in those cells. Expression of the active form of Stat3 protein was assessed with anti-Flag and anti-Stat3 antibodies by Western blotting (Figure 2A). The levels of TF protein were higher in two of the PC14PE6/AS2 cells overexpressing the active form of Stat3 (S3C(1) and S3C(2)) than in the vector control cells (Vec(1)) (Figure 2A). Two clones of PC14PE6/AS2-Stat3D cells stably expressing the dominant negative mutant form of Stat3 (Stat3D) were selected. Expression of Stat3D protein was confirmed using Western blots probed with anti-HA or anti-Stat3 antibodies. The protein levels of TF were markedly lower in the two clones PC14PE6/AS2-Stat3D, S3D(1) and S3D(2), as compared with those of vector control cells (Vec(1)) (Figure 2B). Since TF is expressed in the cell membrane, we next determined the effects of Stat3 activation on the cell surface expression of TF. Cells without permeabilization were fixed and immunofluorescent stained with TF and visualized by confocal microscopy. Figure 2C shows TF expression was indeed expressed on cell surface and markedly decreased by Stat3D in PC14PE6/AS2 cells. Flow cytometry was used to further confirm the TF expression on cell surface. As expected, the two clones PC14PE6/AS2-S3D (S3D(1) and S3D(2)) showed lower TF expression on their cell membranes than vector control cells (Vec(1) and Vec(2)) (Figure 2D). Taken together, our data clearly demonstrate that autocrine IL-6-induced Stat3 activation regulates membrane TF expression in lung cancer cells.

**Figure 3:**
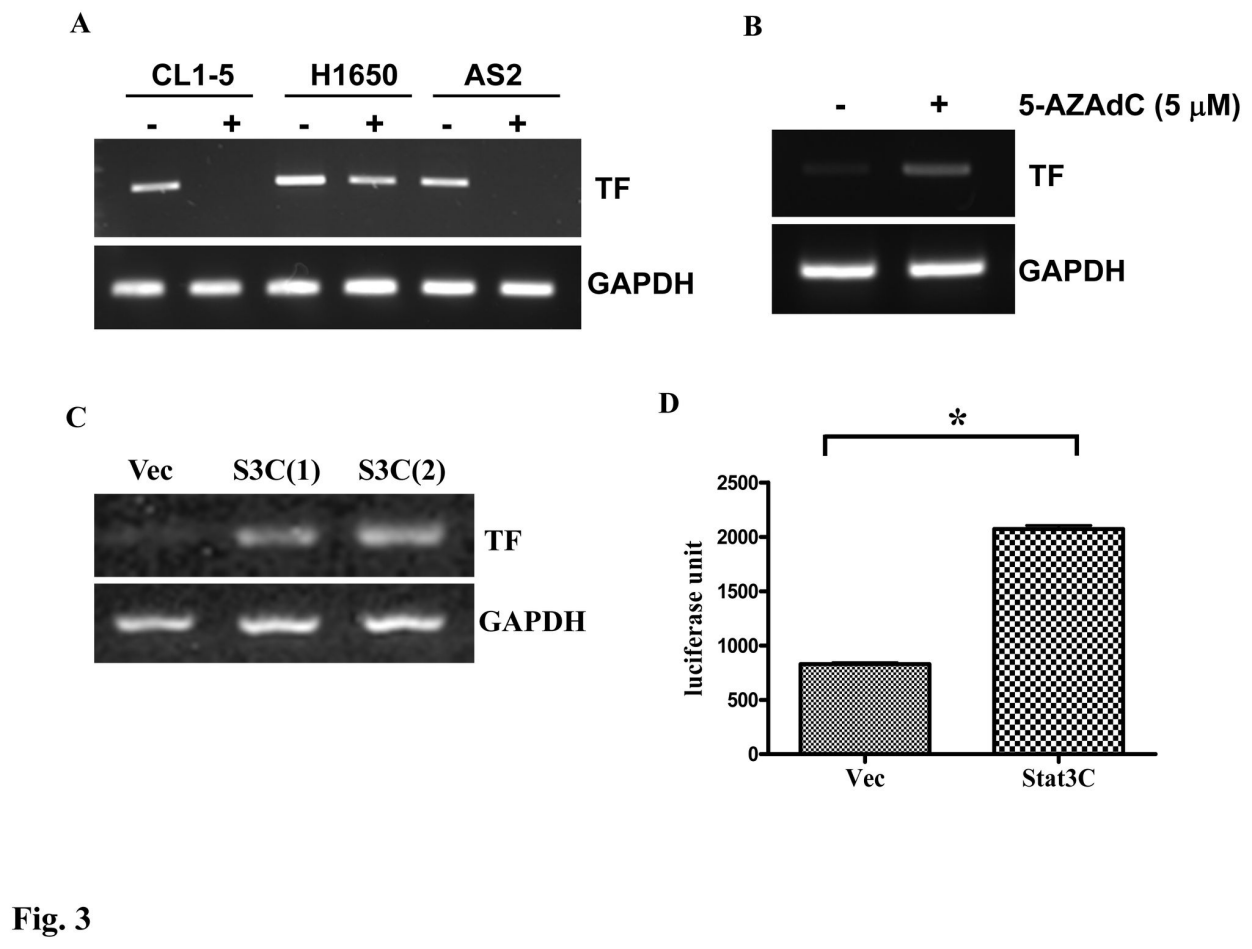
Stat3 activation regulates TF expression at mRNA level in lung cancer cells. (A) CL 1-5, H1650 and AS2 lung adenocarcinoma cells were treated with the JAK inhibitor, INC424 (2.5 µM), TF mRNA levels were analyzed by RT-PCR. (B) CL 1-0 cells were treated with 5-AZAdC (5 µM) everyday for three days and mRNA was prepared for examination of TF expression. (C) mRNA of PC14PE6/AS2-Vec, S3C(1), S3C(2) cells was extracted and subjected to RT-PCR analysis using TF and GAPDH primers. (D) PC14PE6/AS2 cells were transiently transfected with TF promoter reporter gene and active form Stat3 (Stat3C) plasmids or vector control plasmid (Vec). The luciferase activity was determined by luciferase assay system 24 hr after transfection.

We used RT-PCR assay to show that TF mRNA levels were reduced by the treatment of INC424 in CL 1-5, H1650 and PC14PE6/AS2 (AS2) cells (Figure 3A). In AS2 derivative with active form Stat3-Stat3C cells, we found that the TF mRNA expression (Figure 3C) and the promoter activity of TF (Figure 3D) are upregulated. The data further confirm the correlation between Stat3 and TF expression. Because CL 1-0 and CL 1-5 cells are isogenetic, we suspect that the discrepancy between their TF expression may be caused by epigenetic change. We therefore had treated CL 1-0 cells with 5-aza-deoxycytidine (5-AZAdC) and we found TF mRNA levels are induced by 5-AZAdC treatment, indicating that the very little TF in CL 1-0 cells may be due to DNA methylation on TF promoter (Figure 3B). Altogether, our data showed that Stat3 is a direct regulator of TF mRNA expression.

### Blockage of Stat3 activation decreased TF activity and tumor formation of lung cancer cells

**Figure 4:**
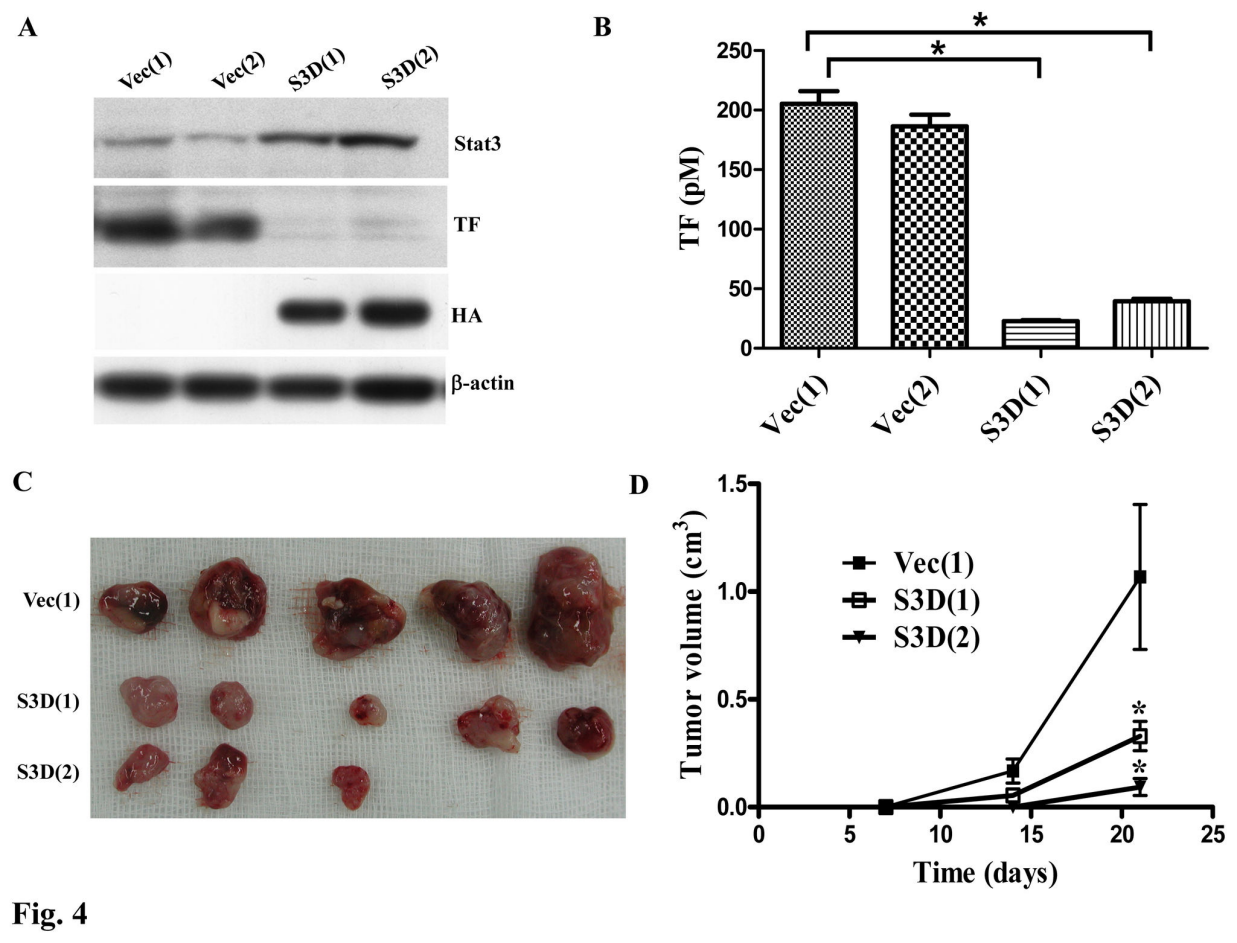
Blockage of Stat3 activation decreased TF activity and tumor formation *in vivo*. (A) Before injecting the cells into nude mice, Vec(1), Vec(2), S3C(1) and S3C(2) cells were harvested and the expression levels of TF and Stat3 were evaluated by Western blot analysis. (B) The same cells as (A) were harvested and TF activity assay were performed to measure the ability to initiate the coagulation cascade. (C) Nude mice were subcutaneously injected with 1 × 10^6^ cells of the vector control clone Vec(1) or dominant-negative Stat3 clones, S3D (1) and S3D(2). Tumor volume was measured weekly. The values of tumor volume represent an average of five mice for each group. *Significantly different from vector controls (P < 0.05) at indicated times (D).

Stat3 is an important oncogene in various human cancers [33] and the TF-activated coagulation is required for primary tumor growth [15]. We have shown that TF expression is regulated by Stat3, and therefore, the effects of Stat3 activation on TF activity and tumor formation were evaluated. Before subcutaneously injecting the cells into the nude mice, the expression of Stat3 and TF and TF-induced coagulation of the cells were evaluated. Again, dominant negative Stat3 (S3D) suppression of TF expression of the cells was detected (Figure 4A). The TF-induced coagulation was also decreased by inhibition of Stat3 activation with S3D, as demonstrated by measuring the ability of TF/FVIIa to activate factor X (FX) to factor Xa (Figure 4B). To reveal the relationship between TF activity and tumor growth, the tumorigenicity of the PC14PE6/AS2 derivatives were evaluated by subcutaneously injecting the cells into the nude mice. The results showed that S3D(1) and S3D(2) cells exhibited lower tumorigenicity than those of Vec(1) cells, indicating that inhibition of Stat3 activation reduced tumorigenicity of the PC14PE6/AS2 cells, possibly resulting in decreased coagulation induced by TF (Figure 4C and D).

### Inhibition of Stat3 activation or downregulation of TF reduced anchorage-independent cell growth in lung cancer cells

**Figure 5:**
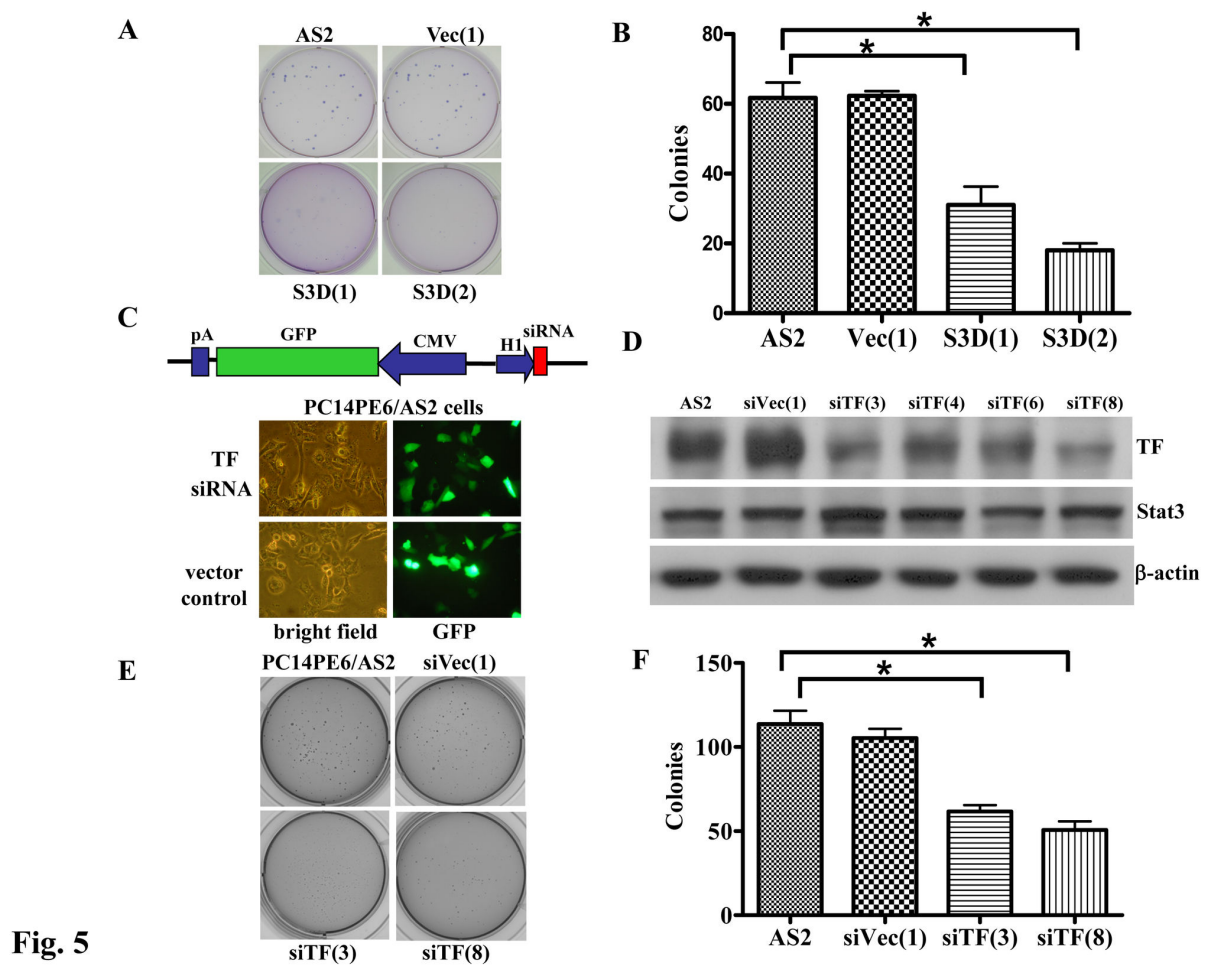
Blockage of Stat3 activation or knockdown of TF expression decreased colony formation in PC14PE6/AS2 cells. (A) Anchorage-independent growth was assessed by colony formation in soft agar. PC14PE6/AS2 parental cells (AS2), Vec (1), S3D(1), and S3D(2) cells were seeded at 1 × 10^3^ cells per well. Colonies were scored 14 days after plating. Experiments were performed in triplicate. Data represent mean ± SEM of three independent experiments. (B) The number of colonies of three repeats was quantified. (C) Upper panel: TF siRNA plasmid contains H1 promoter, which drives TF RNAi expression. CMV promoter drives GFP marker gene expression. Lower panel: PC14PE6/AS2 cells were transiently transfected with TF siRNA (siTF) or vector control plasmids (siVec). GFP expression was detected by fluorescent microscopy and photographed 72 h after transfection. (D) PC14PE6/AS2 cells were transfected with TF siRNA or control plasmids. Stable cell lines were established and the lysates were analyzed by Western blotting using anti-TF antibody. (E) and (F) show anchorage-independent growth of PC14PE6/AS2, siVec(1), siTF(3) and siTF(8) cells. The same procedure was conducted as described in (A) and (B).

Cell’s ability to form tumors *in vivo* correlates with their anchorage dependency *in vitro*. Stat3-transformed cells gain the ability to form colonies in soft agar [27], and inhibition of TF signaling suppresses tumor growth [15]. Therefore, the effect of Stat3 and TF expression on anchorage-independent growth was evaluated. Figure 5A and B shows that the abilities of anchorage-independent growth of S3D(1) and S3D(2) cells, but not vector control (Vec(1)) cells were decreased by dominant negative Stat3 compared to those of the parental cells (AS2). To show that TF acts downstream of Stat3 and contributes to the anchorage-independent growth of PC14PE6/AS2 cells, we constructed specific siRNA plasmids to knock down the expression of TF protein in the cells. TF siRNA expression is under the control of the H1 promoter and GFP expression is driven by a CMV promoter (Figure 5C, upper panel). GFP expression was detected using fluorescence microscopy (Figure 5C, lower panel) in either TF knockdown stable cell lines (siTF) or vector control cells (siVec). Four stable clones showed the specific knockdown effects on TF protein as demonstrated by Western blot analysis (Figure 5D). The levels of Stat3 protein were unaffected by decreased TF expression. The clones, siTF(3) and siTF(8), express less TF and have less anchorage-independent growth potential as compared to the parental (PC14PE6/AS2) cells or vector control cells (siVec(1)) (Figure 5E and F). Together, our data showed that inhibition of Stat3 activation or knockdown of TF expression in lung cancer cells decreased colony formation ability.

### Suppression of TF expression reduced cell adhesion, tumor metastasis and PE formation In Vivo

**Figure 6:**
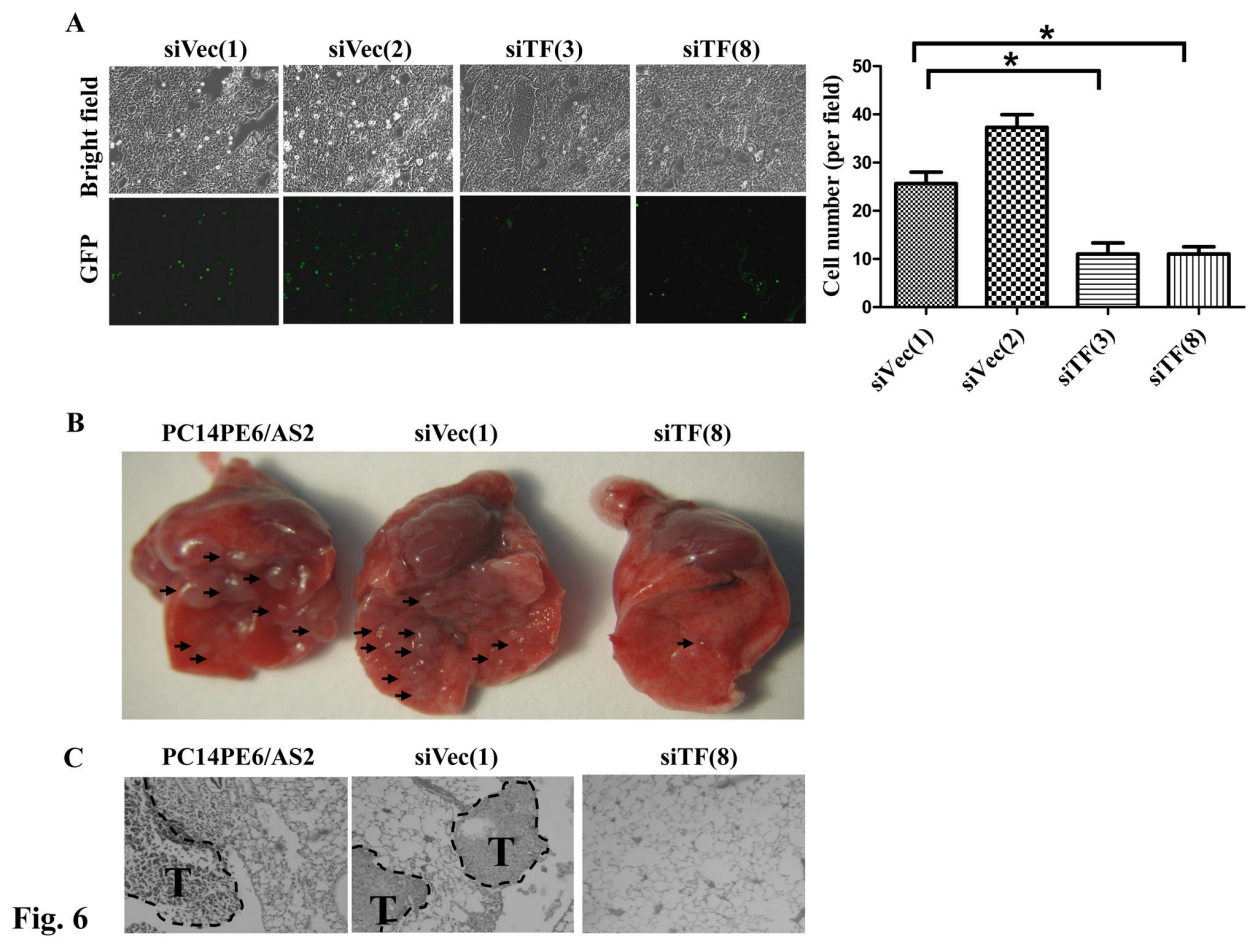
Blockage of TF expression decreased cell adhesion and lung metastasis in nude mice. (A) PC14PE6/AS2-siTF (siTF(3) and siTF(8) and PC14PE6/AS2-siVec cells (siVec(1) and siVec(2)) stably expressing Green fluorescent protein (GFP) were applied to the frozen lung sections. The glass slides were shocked at 70 rpm for 20 min. After PBS wash, adhering cells were fixed and photographed by fluorescent microscopy (left panel). The cell number was quantified as right panel. (B) Various cells (1 × 10^6^) such as parental (PC14PE6/AS2), vector control (siVec(1)), or siTF-transfected PC14PE6/AS2 cells (siTF(8)) were suspended in 0.1 ml PBS and then injected intravenously into the tail veins of nude mice. Mice were sacrificed and lungs were excised and photographed 26 days after injection. Block arrows: metastatic tumor nodules. (C) Histological analysis of lung metastasis of PC14PE6/AS2, siVec(1) or siTF(8) cells. Paraffin-embedded lung tissues were sectioned into 4 µm thick sections, and then stained with hematoxylin-eosin. Metastatic tumors (T) are shown within the PC14PE6/AS2 and siVec(1) lung tissue. The siTF(8) tumor had no foci.

Coagulation facilitates the spread of tumor cells in the pulmonary vasculature during early metastatic colony formation [34], and TF promotes cell migration and spreading via interacting with integrin α3β1 [35]; therefore, the effects of silencing TF expression on cell adhesion *in vivo* were assessed. The Stamper-Woodruff assay was used to evaluate the adhesion ability of PC14PE6/AS2-siTF (siTF(3) and siTF(8)) and PC14PE6/AS2-siVec (siVec(1) and siVec(2)) cells stably expressing GFP to normal lung tissues. Figure 6A shows that while TF expression was silenced, the ability of these cells to adhere to the lung tissue was suppressed, suggesting that TF expression participated in early metastatic colony formation.

We previously found constitutively activated Stat3 promotes tumor metastasis of lung adenocarcinoma [21]; therefore, we sought to investigate the role of TF in lung metastasis subsequently. PC14PE6/AS2, siVec(1) and siTF(8) cells were individually injected into the tail veins of nude mice. The incidence of lung metastasis in mice injected with siTF(8) was significantly lower than in those injected with parental PC14PE6/AS2 or vector control (siVec(1)) cells. The number of lesions in mice with lung metastasis was also significantly lower in mice injected with siTF(8) cells than in mice injected with PC14PE6/AS2 or siVec(1) cells. Accordingly, none of the mice injected with siTF(8) developed pleural effusion (PE), but 3 of 4 and 4 of 4 mice injected with PC14PE6/AS2 or siVec(1) developed PE (Figure 6B and Table 1). Altogether, our data indicate that suppression of Stat3-induced TF expression in lung cancer cells decreased colony formation *in vitro*, as well as cell adhesion, lung metastasis *in vivo*.

**Table 1:** Production of experimental metastasis and pleural effusion (PE) by PC14PE6/AS2 cells transfected with TF siRNA vector in nude mice.

	**lung lesions**		**PE volume (ml)**	
**Cell lines**	**Incidence**	**Average (range)**	**Incidence**	**Average (range)**
PC14PE6/AS2	4/4	30.5 (2-56)	3/4	0.32 (0-0.64)
siVec(1)	4/4	32.5 (7-47)	4/4	0.09 (0.01-0.15)
siTF(8)	1/4	0.25 (0-1)	0/4	0

Tumor cells (1 x 10^6^) were injected intravenously (i.v.) into nude mice. The experiment was terminated on day 26, the mice development of lung lesions and PE were evaluated.

### Downregulation of TF by siRNA reduced vascular permeability and ascites formation

**Figure 7:**
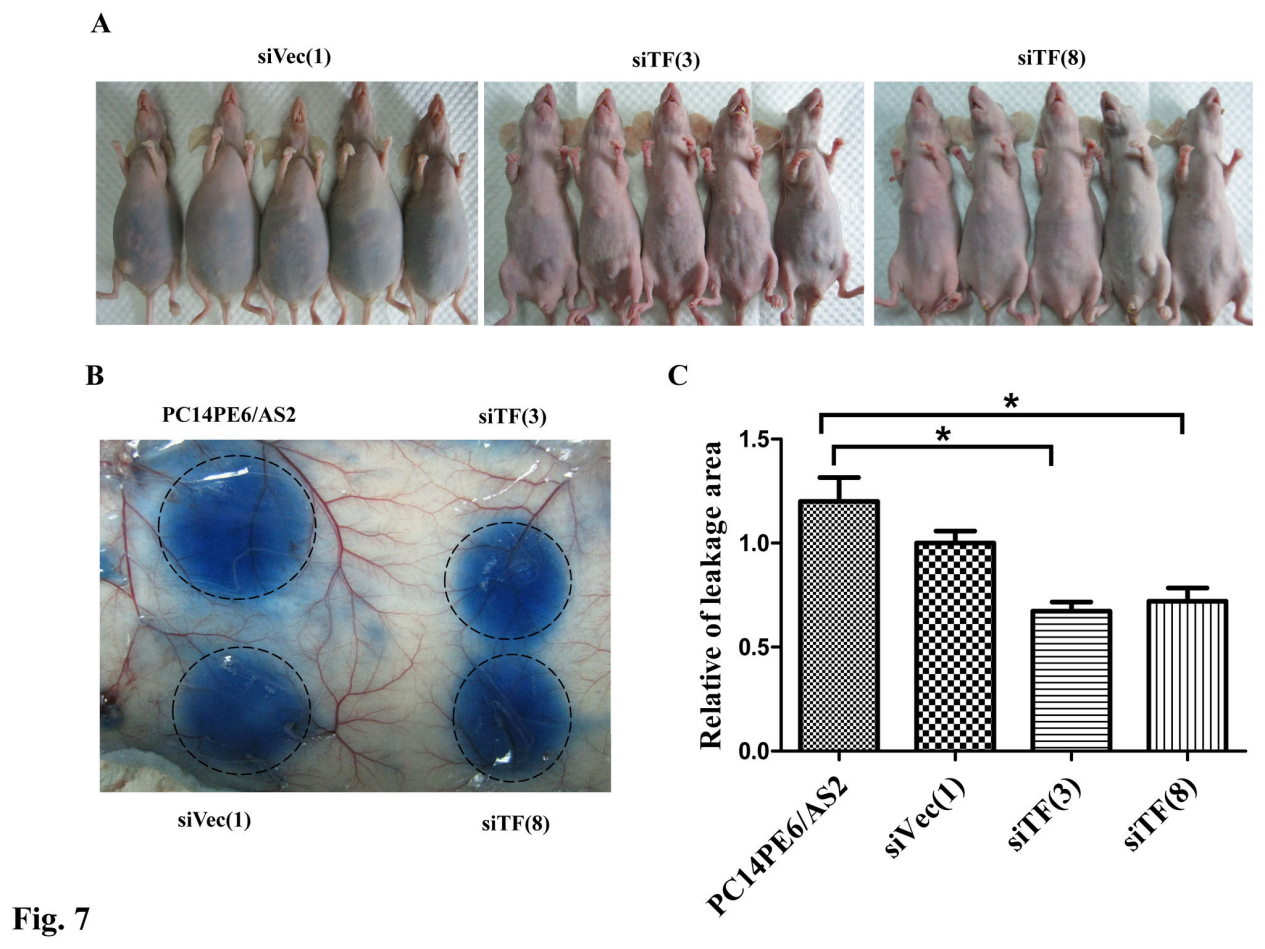
Blockage of TF expression in PC14PE6/AS2 cells decreased ability of vascular permeability and ascites formation. (A) Gross appearance of nude mice showed abdominal distension with dark fluid after 21 days in mouse injected with siVec(1) cells, but not in two other groups of mice injected with siTF(3) and siTF(8) cells. (B) Serum-free culture supernatants of tumor cells were harvested for the Miles assay. Nude mice were intravenously injected with Evans blue dye. After 10 min, 50 µl of test samples (from PC14PE6/AS2, siVec(1), siTF(3), or siTF(8) cells) were injected into the dermis of the mice. The subdermis was harvested and photographed after 30 min. Dye leakage areas were indicated by dashed black circles and quantified (C) (n=3).

Because decreased formation of MPE may result from reduced lung metastasis and/or vascular permeability, the effects of TF on vascular permeability were evaluated in the ascites nude mouse model and by the Miles assay. Nude mice were intraperitoneally injected with siVec(1), siTF(3) or siTF(8) cells. Mice injected with siVec(1) cells produced more malignant ascites than siTF(3) and siTF(8) cells (Figure 7A). Malignant ascites developed in 3 of 5 mice with siTF(3) cells (average: 0.11 ml) and 1 of 5 mice with siTF(8) cells (average: 0.02 ml), respectively, and all mice in which siVec(1) cells were injected developed a considerable amount of malignant ascites (range: 3.8-5.3 ml; average: 4.84 ml) (Table 2). The Miles assay was used to further determine the degree of vessel hyperpermeability induced by TF. The areas of dye leakage induced by conditioned medium from siTF(3) and siTF(8) cells but not from siVec(1) cells were significantly smaller than the leakage area from parental PC14PE6/AS2 cells (Figure 7B and C). Collectively, the results clearly indicate that downregulation of TF expression can reduce vascular permeability as well as malignant ascites formation.

**Table 2:** Production of experimental ascites by PC14PE6/AS2 cells transfected with TF siRNA vector in nude mice.

**Cell lines**	**Incidence**	**Ascites volume (ml)**	**Average (ml)**
siVec(1)	5/5	3.8, 5.2, 5.1, 5.3, 4.8	4.84
siTF(3)	3/5	0.5, 0.05, 0.1	0.11
siTF(8)	1/5	0.1	0.02

Tumor cells (1 x 10^6^) were injected into peritoneum (i.p.) into nude mice. The experiment was terminated on day 21, the mice development malignant ascites were evaluated.

## Discussion

Autocrine IL-6-induced Stat3 activation has been implicated in tumor metastasis and the formation of MPE in lung adenocarcinoma [21]. In this study, TF was demonstrated as a Stat3 downstream gene and regulated by JAK2 signaling. Expression of TF in lung adenocarcinoma cells increased formation of colonies *in vitro*, and cell adhesion as well as lung metastasis *in vivo*. Moreover, TF expression increased vascular permeability to promote MPE formation in lung adenocarcinoma. Together, we found that the expression of TF regulated by IL-6/JAK2/Stat3 signaling promotes tumor metastasis and MPE formation in an animal model of lung adenocarcinoma.

TF is highly expressed in various cancers, including lung cancer [36]. Although TF functions as a coagulation factor, it is also associated with tumor growth, angiogenesis, as well as metastasis [37]. Blockage of TF signaling using anti-TF antibodies disrupts tumor growth; and inhibition of TF-induced protease-activated receptor 2 (PAR2) suppresses experimental lung metastasis of breast cancer cells [15]. Moreover, silencing of TF by siRNA *ex vivo* or *in vivo* also inhibits experimental lung metastasis of B16 melanoma cells [38]. In our study, the regulation of TF by IL-6/JAK2/Stat3 signaling, which participates in metastasis, was also confirmed in lung cancer cells. Briefly, the stable cell lines PC14PE6/AS2 in which TF has been silenced by siRNA produced fewer nodules in the lungs as compared to the vector control cell lines. Therefore, the TF-activated coagulation cascade in the tumor microenvironment was developed as an effective target for cancer therapy [25]. TF constitutive association with α3β1 integrin in breast cancer cells is known to promote tumor metastasis [15]. It has also been reported that coagulation facilitates tumor cell spread in the premetastatic niche of the pulmonary vasculature during early metastatic colony formation [34]. Moreover, TF-induced clot formation by tumor cells indirectly enhances tumor cell survival via macrophage recruitment in the lungs in the early stages of the metastatic process [39]. We also demonstrated using the Stamper-Woodruff assay that Stat3-induced TF expression promotes tumor cell adhesion to lung tissues. However, whether TF interacts directly with α3β1 integrin in this study needs to be further clarified. Previously, we found that autocrine IL-6-induced Stat3 activation contributes to tumor metastasis of lung adenocarcinoma [21]. In this study, we showed that inhibition of Stat3 activation resulted in decreased coagulation induced by TF. Furthermore, knockdown of TF expression decreased experimental lung metastasis. Taken together, TF may contribute to Stat3 activation-induced tumor metastasis via coagulation in lung cancer cells.

TF is detected highly expressed in MPE [17] and is able to increase permeability of the microvasculature, which could be a major causative factor in the induction of MPE [26]. Using a mouse model with intravenous injections of PC14PE6/AS2 lung adenocarcinoma cells, we have found that knockdown of TF by siRNA in PC14PE6/AS2 cells produced less MPE. Recent reports have shown that cancer cell surface expression of the TF:FVIa complex activates coagulation within the tumor microenvironment, which enhanced permeability of tumor microenvironment [25]. In our study, we showed that surface TF expression was regulated by IL-6/Stat3 signaling, and TF-induced coagulation was decreased by inhibiting Stat3 activation in lung cancer cells. We also showed that knockdown of TF expression decreased vascular permeability in the Miles assay. Moreover, less ascites was produced by knockdown of TF expression in the nude mouse model. Altogether, TF was clearly shown to be regulated by IL-6/Stat3 signaling and involved in the formation of lung adenocarcinoma-associated MPE through modulation of vascular permeability in the tumor microenvironment. Using the nude mouse model, we previously reported that VEGF is another downstream target of Stat3, and the expression of VEGF increases vascular permeability and causes MPE [21]. One report showed that the expression of VEGF is regulated by TF [40]. In this study, however, VEGF expression was not altered by knockdown of TF expression in PC14PE6/AS2 lung cancer cells (Figure S2). Taken together, these findings indicate that, in lung adenocarcinoma bearing activated Stat3, MPE generation can be independently induced by TF or VEGF.

Various oncogenes alter TF expression and convert the tumor phenotype from noninvasive to invasive [41-44]. c-Met activation induced TF expression leads to cell migration in medulloblastoma [42]. Overexpression of EGFR in A431 human carcinoma cells and glioma cells causes increased TF expression which functions in tumor initiation and angiogenesis [41]. Activation of K-ras and loss of p53 are involved in TF regulation, which is an important factor in the induction of K-ras-dependent tumorigenesis and angiogenesis in colorectal carcinoma [43]. In non-small cell lung cancer, increased TF expression is also associated with worse survival and with mutations of TP53 and PTEN [12]. In this study, we demonstrated that TF expression was regulated by IL-6/JAK2/Stat3 signaling by pharmaceutical inhibition of JAKs and suppressed Stat3 activation using a genetic approach. Our results indicate IL-6/JAK2/Stat3 pathway also regulates TF expression, which is an important regulator for tumor formation, lung metastasis, and malignant effusion generation in lung adenocarcinoma bearing activated Stat3.

Several transcription factors bind to the TF promoter and regulate TF promoter activity under various conditions [45]. For example, early growth response gene-1 (EGR-1) regulates TF expression under hypoxic conditions in glioblastoma multiforme [46]. EGFR and PTEN modulate TF expression through JunD/AP-1 in glioblastoma [44]. In the human endometrium, TF expression could be regulated through Sp1 and Sp3 binding on the promoter [47]. In our study, the regulation by the Stat3 transcription factor on TF expression was demonstrated by overexpression of active or dominant negative forms of Stat3 in PC14PE6/AS2 cells. We showed that Stat3 is a direct regulator of TF mRNA expression, supported by data from RT-PCR and reporter gene assay (Figure 3). However, whether Stat3 binds directly to TF promoter needs to be further investigated. Furthermore, TF promoter activity was increased by expression of the active form of Stat3 in PC14PE6/AS2 cells. However, it is still unclear whether Stat3 binds directly to the TF promoter and cooperates with other factors to regulate TF expression. These mechanisms therefore warrant further investigation.

In conclusion, we demonstrate that TF is regulated by Stat3 activation and is an important regulator for the generation of MPE in lung adenocarcinoma. Targeting TF and VEGF together may be necessary for the control of MPE in lung cancer patients.

## Supporting Information

Figure S1 **Effects of PP3 on Stat3 activation and TF expression in PC14PE6/AS2 cells.** PC14PE6/AS2 cells, after seeding for 24 hr, were incubated in the serum-free medium without (−) or with (+) PP3 (40 µM) for the indicated times. Cell lysates were analyzed by Western blot analysis using various antibodies as indicated.
(TIF)

Figure S2 **Knockdown of TF expression does not alter VEGF expression.** VEGF levels in the culture medium of PC14PE6/AS2, siVec(1), siTF(1) and siTF(2) cells after being plated for 24 h were measured using ELISA.
(TIF)
