# Supplementary figures and images for: Upregulation of Tissue Factor by Activated Stat3 Contributes to Malignant Pleural Effusion Generation via Enhancing Tumor Metastasis and Vascular Permeability in Lung Adenocarcinoma
Source: PLoS One. 2013 Sep 27;8(9):e75287. doi: 10.1371/journal.pone.0075287 (PMC3785526; doi:10.1371/journal.pone.0075287)

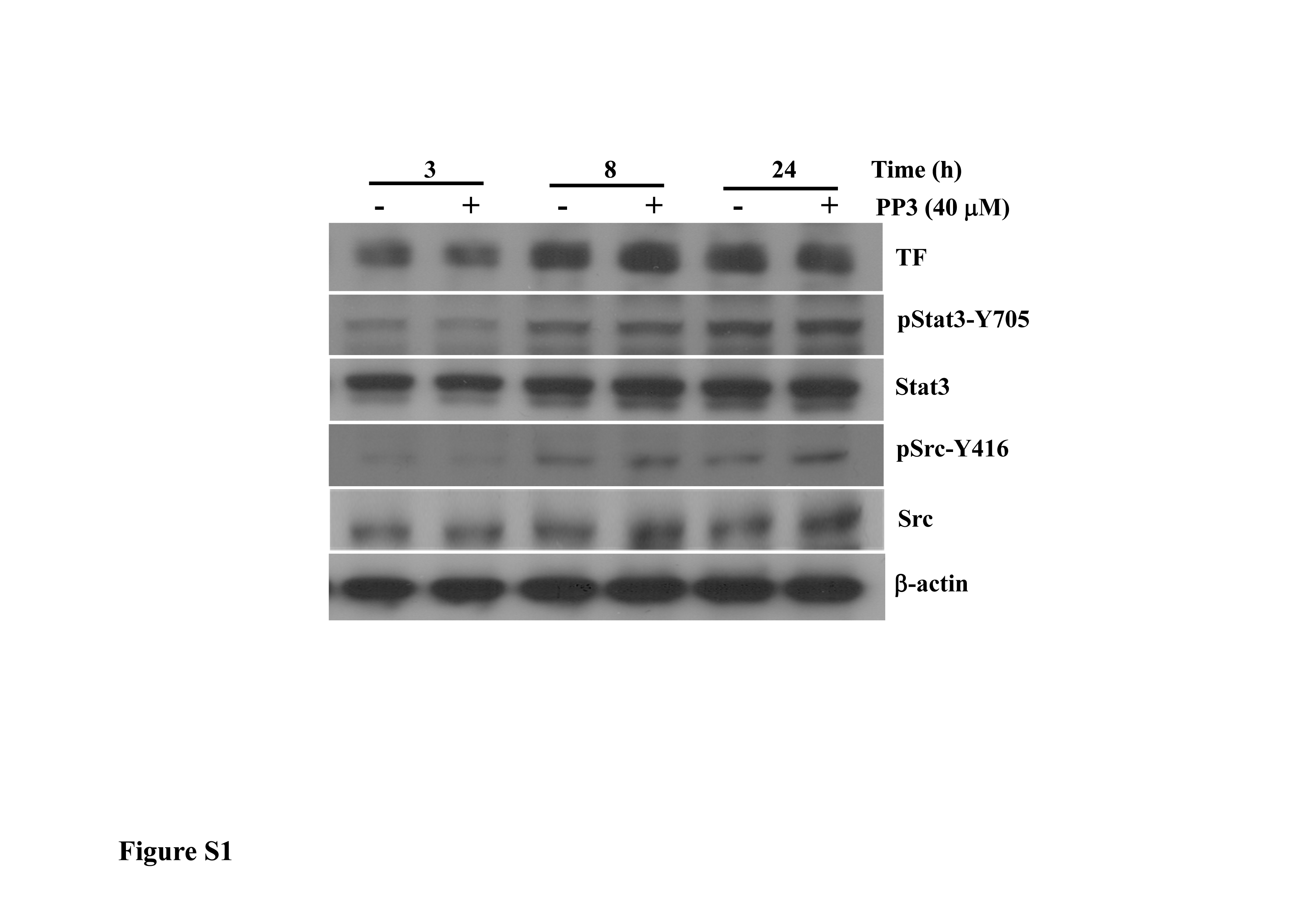

Supplement: Figure S1 — Effects of PP3 on Stat3 activation and TF expression in PC14PE6/AS2 cells. PC14PE6/AS2 cells, after seeding for 24 hr, were incubated in the serum-free medium without (−) or with (+) PP3 (40 µM) for the indicated times. Cell lysates were analyzed by Western blot analysis using various antibodies as indicated. (TIF) [file pone.0075287.s001.tif]

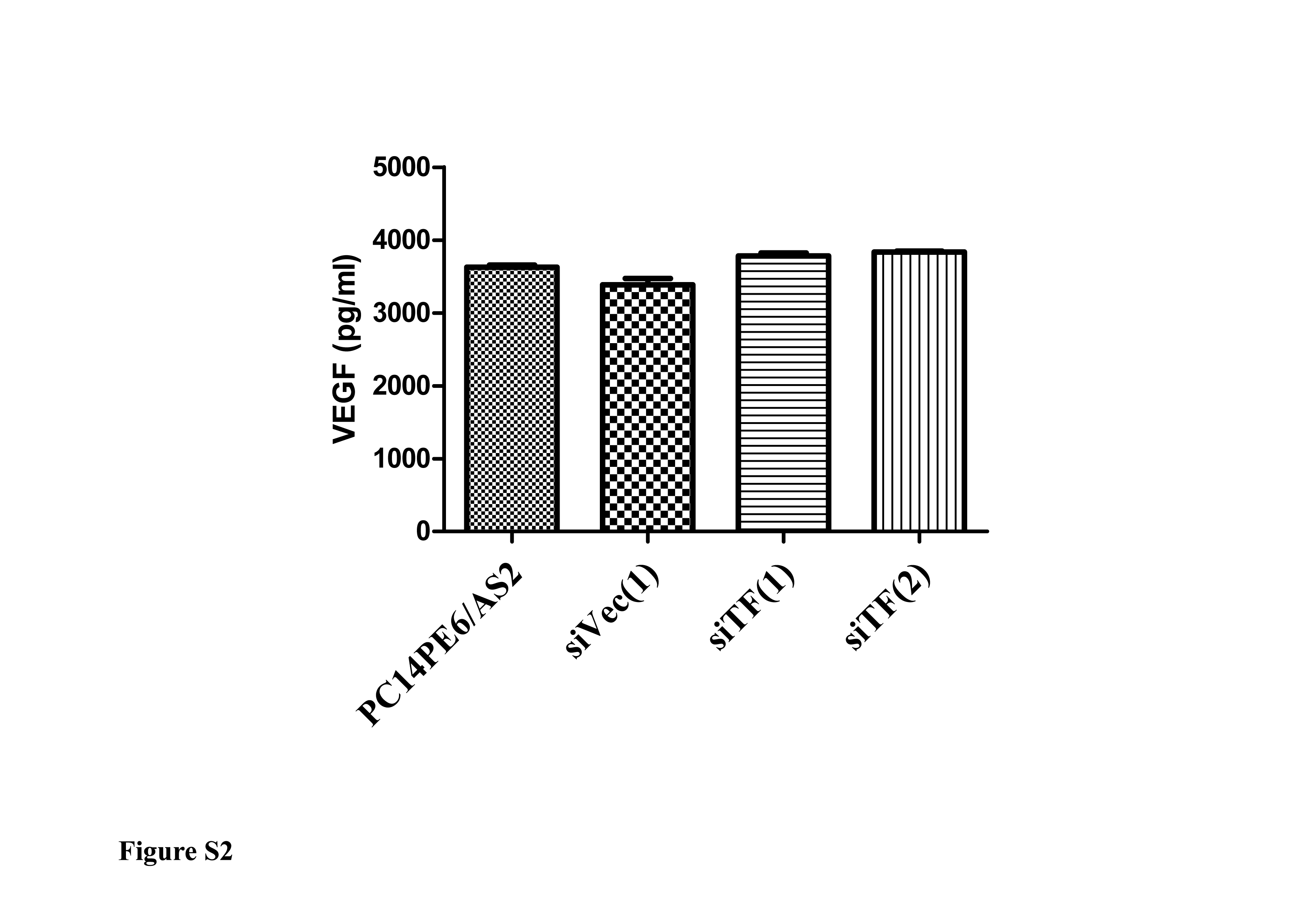

Supplement: Figure S2 — Knockdown of TF expression does not alter VEGF expression. VEGF levels in the culture medium of PC14PE6/AS2, siVec(1), siTF(1) and siTF(2) cells after being plated for 24 h were measured using ELISA. (TIF) [file pone.0075287.s002.tif]
